# A Fourth *KLK4* Mutation Is Associated with Enamel Hypomineralisation and Structural Abnormalities

**DOI:** 10.3389/fphys.2017.00333

**Published:** 2017-05-29

**Authors:** Claire E. L. Smith, Jennifer Kirkham, Peter F. Day, Francesca Soldani, Esther J. McDerra, James A. Poulter, Christopher F. Inglehearn, Alan J. Mighell, Steven J. Brookes

**Affiliations:** ^1^Department of Oral Biology, School of Dentistry, St James's University Hospital, University of LeedsLeeds, United Kingdom; ^2^Section of Ophthalmology and Neuroscience, St James's University Hospital, University of LeedsLeeds, United Kingdom; ^3^School of Dentistry, University of LeedsLeeds, United Kingdom; ^4^Bradford District Care NHS Foundation Trust, Community Dental Service, Horton Park Health CentreBradford, United Kingdom; ^5^Locala Dental Care, Dental Department, Batley Health CentreBatley, United Kingdom

**Keywords:** KLK4, enamel, phenotype, mutation, amelogenesis imperfecta

## Abstract

“Amelogenesis imperfecta” (AI) describes a group of genetic conditions that result in defects in tooth enamel formation. Mutations in many genes are known to cause AI, including the gene encoding the serine protease, kallikrein related peptidase 4 (*KLK4*), expressed during the maturation stage of amelogenesis. In this study we report the fourth *KLK4* mutation to be identified in autosomal recessively-inherited hypomaturation type AI, c.632delT, p.(L211Rfs^*^37) (NM_004917.4, NP_004908.4). This homozygous variant was identified in five Pakistani AI families and is predicted to result in a transcript with a premature stop codon that escapes nonsense mediated decay. However, the protein may misfold, as three of six disulphide bonds would be disrupted, and may be degraded or non-functional as a result. Primary teeth were obtained from one affected individual. The enamel phenotype was characterized using high-resolution computerized X-ray tomography (CT), scanning electron microscopy (SEM), energy dispersive X-ray spectroscopy (EDX), and microhardness testing (MH). Enamel from the affected individual (referred to as KLK4 enamel) was hypomineralised in comparison with matched control enamel. Furthermore, KLK4 inner enamel was hypomineralised compared with KLK4 outer enamel. SEM showed a clear structural demarcation between KLK4 inner and outer enamel, although enamel structure was similar to control tissue overall. EDX showed that KLK4 inner enamel contained less calcium and phosphorus and more nitrogen than control inner enamel and KLK4 outer enamel. MH testing showed that KLK4 inner enamel was significantly softer than KLK4 outer enamel (*p* < 0.001). However, the hardness of control inner enamel was not significantly different to that of control outer enamel. Overall, these findings suggest that the *KLK4* c.632delT mutation may be a common cause of autosomal recessive AI in the Pakistani population. The phenotype data obtained mirror findings in the *Klk4*^−/−^ mouse and suggest that KLK4 is required for the hardening and mineralization of the inner enamel layer but is less essential for hardening and mineralization of the outer enamel layer.

## Introduction

Formation of the complex structure of tooth enamel is remarkable. Enamel is both extremely hard and very dense. These properties enable tooth enamel to withstand the large forces experienced during mastication and make it the hardest tissue in the human body.

Amelogenesis, the process of enamel formation, can be broadly subdivided into three stages: secretion, transition and maturation. During secretion, an extracellular matrix (ECM) of enamel proteins, including amelogenin (AMELX as well as AMELY for males), enamelin (ENAM), and ameloblastin (AMBN), is secreted by the enamel secreting cells, the ameloblasts. Except for initial and final layers of enamel secreted, the bulk of the enamel is secreted via an apical cellular extension on each ameloblast, termed the Tomes' process. During secretion, the ameloblasts retreat away from the forming dentine to produce a layer of enamel matrix containing immature enamel crystals that span the full thickness of the future enamel that are organized into rod and inter rod enamel (Skobe, 1976). Following their secretion, enamel proteins are specifically cleaved by the secretory stage proteinase, matrix metallopeptidase 20 (MMP20), to produce peptides with seemingly specific roles in enamel formation (Simmer and Hu, 2002). During the transition stage, the ameloblasts reduce secretion of enamel proteins and MMP20, lose their Tomes' processes and undergo phenotypic remodeling, reflecting their reduced rate of secretion (Reith, 1970). At this stage, the ameloblasts also begin to secrete the enamel serine protease, kallikrein related peptidase 4 (KLK4), which reaches maximal secretion during the maturation stage (Hu et al., 2000, 2002). The maturation stage involves the complete breakdown of the enamel protein matrix and its removal, concurrent with secondary mineralization of the enamel ECM. KLK4 acts to degrade the remaining enamel matrix peptides to facilitate their removal by endocytosis. The removal of matrix protein from the forming enamel and its replacement with tissue fluid provides space for the enamel crystals to grow in width. At the same time, mineral ions are pumped into the enamel to support mineral growth. Once formed, enamel is around 96% mineral by weight, 3% protein, and 1% water (Deakins and Burt, 1944).

Perturbed amelogenesis can result in amelogenesis imperfecta (AI), a collection of genetic conditions resulting in defective tooth enamel. AI has been broadly classified into hypoplastic and hypomineralised types (Gadhia et al., 2012). Hypoplastic AI results from failure during the secretory stage and is characterized by thin enamel that is variably mineralized. Hypomineralised AI can be further subdivided into hypocalcified and hypomaturation sub-types, where the enamel is soft and brittle, respectively. AI can show autosomal dominant, recessive or X-linked inheritance and is associated with mutations in a number of genes. These include, but are not limited to, genes encoding the enamel proteins, AMELX (Lagerstrom et al., 1991), ENAM (Rajpar et al., 2001), and AMBN (Poulter et al., 2014) as well as the secretory stage metalloproteinase, MMP20 (Kim et al., 2005) and the maturation stage serine proteinase, KLK4 (Hart et al., 2004).

Mutations in *KLK4* have been shown to result in recessively-inherited hypomaturation AI (MIM #204700) (Hart et al., 2004). To date, only three *KLK4* mutations have been reported in a total of four families (Hart et al., 2004; Wright et al., 2011; Wang et al., 2013; Seymen et al., 2015), suggesting that *KLK4* mutations are a relatively rare cause of AI. Mutations described to date include a frameshift and a nonsense mutation for which the resulting transcript is likely to be subject to nonsense mediated decay (NMD). The most recently reported mutation was a frameshift in the final exon, predicted to escape NMD. However, *in vitro* expression showed that a lower level of protein was produced with a lower level of enzymatic activity compared to controls. The human enamel phenotype resulting from *klk4* mutations remains uncharacterized outside of the mouth, but a *Klk4* knockout mouse model (*Klk4*^−/−^) has been extensively characterized (Simmer et al., 2009, 2011; Smith et al., 2011; Yamakoshi et al., 2011; Nunez et al., 2016). The enamel was reported to be of a similar thickness and decussating structure to wild-type (WT) controls but was hypomineralised (Simmer et al., 2009), at levels of around 80 % compared with WT enamel (Smith et al., 2011). The defect was shown to be more severe in the inner enamel layer [toward the dentino-enamel junction (DEJ)] compared with the outer enamel (toward the surface) in these animals, suggesting that KLK4 is needed more for the mineralization of inner enamel than outer enamel (Smith et al., 2011). Microhardness values also mirrored these findings, with softer enamel overall for *Klk4*^−/−^ mice compared with WT, but increasingly soft enamel from the surface to the DEJ (Nunez et al., 2016).

Here we report the fourth *KLK4* mutation, identified in five UK families of Pakistani origin, and characterize the phenotype of the enamel of teeth from one individual.

## Materials and methods

### Patients

Individuals from each of the five families were recruited following informed consent in accordance with the principles outlined in the declaration of Helsinki and with local ethical approval. Genomic DNA was obtained from saliva using Oragene® DNA Sample Collection kits (DNA Genotek, ONT, Canada) according to the manufacturer's instructions. Deciduous teeth were obtained following natural exfoliation.

### Genotyping

#### Whole-exome sequencing and analysis

**Figure 1:**
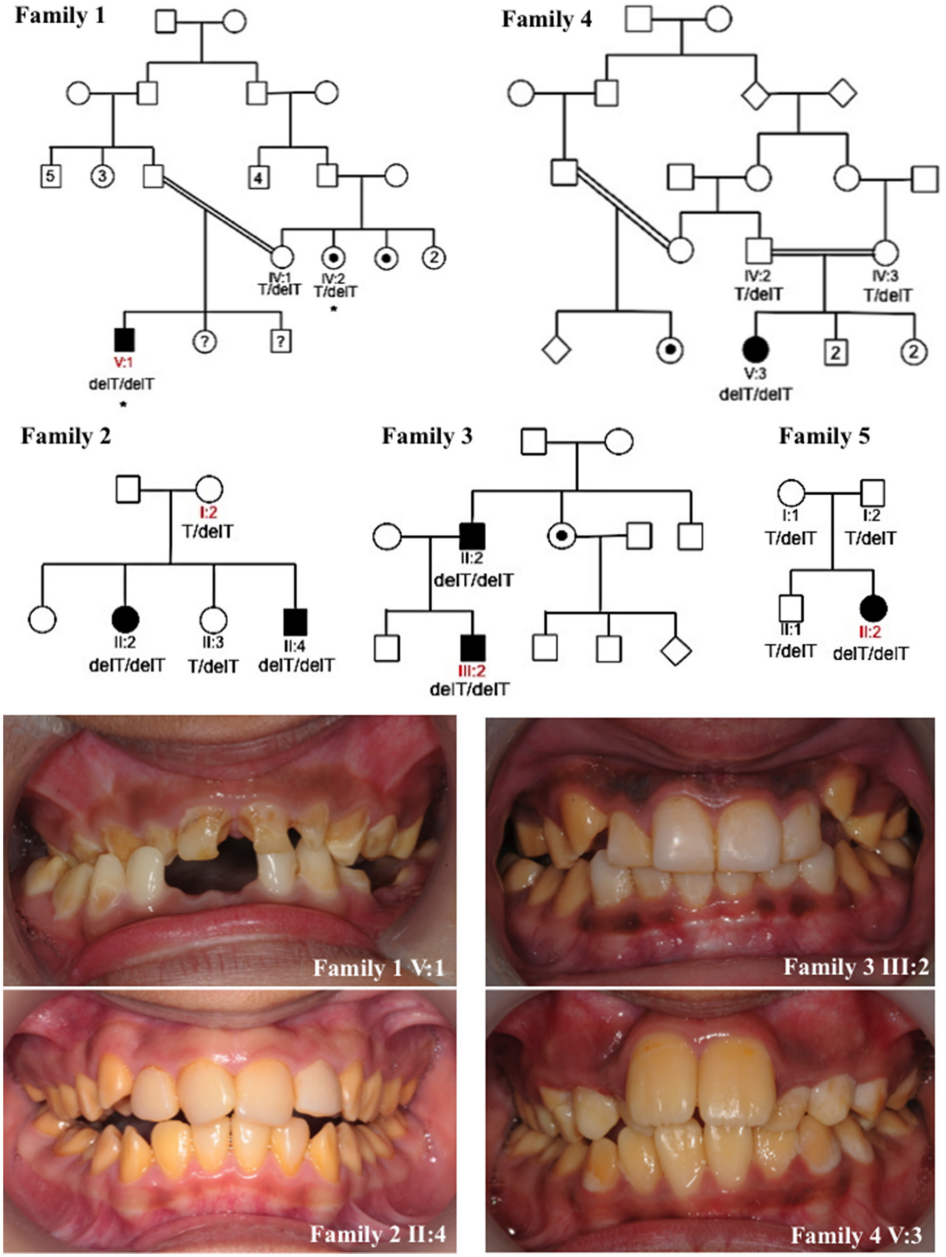
**Pedigrees and clinical images of families 1–5**. Red labels indicate the individuals for which DNA was subjected to whole exome sequencing for families 1–3 and 5. Asterisks mark individuals in family 1 whose DNA underwent SNP genotyping analysis. Dots indicate individuals that self-reported as affected with AI or were reported by other family members to be affected with AI. However these individuals were not themselves clinically assessed by a dental practitioner. Question marks indicate individuals for which details of their AI phenotype were unavailable. The genotypes of the *KLK4* c.632delT variant (NM_004917.4) for each individual for which DNA was available for analysis are marked on each pedigree. Clinical images show hypomaturation AI in families 1–4. Clinical images were unavailable for family 5.

Genomic DNA from a single individual from families 1, 2, 3, and 5 (marked with an arrow on each pedigree, Figure 1) was subjected to whole exome sequencing (WES). Three micrograms (families 1, 2, and 3) or 200 nanograms (family 5) of genomic DNA were processed using the Agilent SureSelect XT Library Prep according to the manufacturer's protocol (Agilent Technologies, CA, USA). Sequencing was performed on an Illumina HiSeq 2500 using a 100 bp paired-end protocol (families 1, 2, and 3) or an Illumina HiSeq 3000 using a 150 bp paired-end protocol (family 5). The fastq files were aligned to the human reference genome (GRCh37) using the Burrows Wheeler aligner (Li and Durbin, 2009). The resulting alignment was processed in the SAM/BAM format using the SAMtools, Picard (http://picard.sourceforge.net) and the Genome Analysis Toolkit (GATK) in order to correct alignments around indel sites and mark potential PCR duplicates (McKenna et al., 2010; DePristo et al., 2011).

Indel and single-nucleotide variants were called in the VCF format using the Haplotype Caller function of the GATK program. Using the VCFhacks package (freely available at https://github.com/gantzgraf/vcfhacks) variants present in NCBI's dbSNP142 or the Exome Aggregation Consortium database (ExAC; v0.3.1) with a minor allele frequency (MAF) ≥1% were excluded. The remaining variants were annotated using NCBI's Variant Effect Preditor. Variants with a Combined Annotation Dependent Depletion (CADD) score of ≥15 were prioritized and variants in genes already known to cause AI were highlighted for segregation analysis.

### PCR and Sanger sequencing

Variants identified by WES were confirmed and segregation performed for all available members of each family. For family 4, variants were identified and segregation performed for all available family members by direct Sanger sequencing of the *KLK4* gene. Primer sequences can be found in Supplementary Table 1. Sanger sequencing was performed using the BigDye Terminator v3.1 kit (Life Technologies, CA, USA) according to manufacturer's instructions and resolved on an ABI3130xl sequencer (Life Technologies, CA, USA). Results were analyzed using SeqScape v2.5 (Life Technologies, CA, USA).

#### SNP genotyping

DNA from two affected individuals, IV:2 and V:1 was genotyped using Affymetrix 6.0 SNP microarrays by AROS Applied Biotechnology (Aarhus, Denmark). The resulting birdseed files were annotated and analyzed using SnpViewer (freely available at https://github.com/gantzgraf/snpviewer) to identify shared regions of homozygosity between both the affected individuals.

#### Microsatellite analysis

Standard primers for markers D11S217, D11S902, D11S246, D11S907, D11S1553, D11S397, and D11S601 were used to assess the flanking haplotypes of the *KLK4* gene for families 1–4 (Supplementary Table 2). Products were sized using GeneScan 500 ROX (Life Technologies, CA, USA) according to the manufacturer's instructions and resolved on an ABI3130xl sequencer (Life Technologies, CA, USA). Results were analyzed using Genemapper v4.0 (Life Technologies, CA, USA).

### Enamel phenotyping

#### Computerized X-ray tomography

Teeth were analyzed by high resolution X-ray microCT using a Skyscan 1172 (Bruker, Coventry, UK) operated at 100 kV with a source current of 100 μA and an aluminum/copper filter to reduce beam hardening. CT slices were reconstructed using Skyscan Recon software (Bruker). The CT images were calibrated using a two point standard of hydroxyapatite mineral of known densities [0.25 and 0.75 g/cm^3^ (Bruker)]. Matched control teeth were obtained from the Skeletal Tissues Research Tissue Bank (School of Dentistry, University of Leeds; NRES REC ref: 07/H1306/95+5). These were obtained with written consent from patients attending clinics at Leeds Dental Hospital, and analyzed during the same scan as their test counterpart. Tooth slices of 500 μm, prepared from both control and KLK4 teeth, were also scanned to ensure that whole tooth results were representative.

Calibrated color contour maps of mineral density were generated using ImageJ and the 3D interactive surface plot plugin. Videos were created using CTVox software (Bruker).

### Tooth sectioning

Control and KLK4 teeth were sectioned in similar planes using an Accutom-5 cutter (Struers, Ballerup, Denmark) fitted with a peripheral diamond cutting disc and cooled with minimal water.

### Scanning electron microscopy

After sectioning, the cut edge of the tooth was polished using 600 and 2000 grade carborundum paper (3M, Maplewood, MN, USA), followed by a nail buffer. Sections were etched by immersion in 30 % phosphoric acid, followed by thorough rinsing in excess distilled water and dried overnight under vacuum. Sections were mounted on aluminum stubs and sputter coated with gold using an auto sputter coater (Agar Scientific, Elektron Technology, Stansted, UK). Microstructural analysis was undertaken using a Hitachi S-3400N scanning electron microscope (Hitachi, Tokyo, Japan), fitted with a 123 eV Nano XFlash® Detector 5010 (Bruker) and operated at an accelerating voltage of 20 kV using secondary electron detection.

### Microhardness testing

Microhardness measurements were carried out on incisor and molar teeth halves. Sections were immobilized on glass slides (cut side up) using superglue adhesive (Loctite, Düsseldorf, Germany). Sections were polished plano-parallel with 1000 grade then 2000 grade carborundum paper (3M, Maplewood, MN, USA) and then finely polished with 5 μm aluminum oxide suspension (Struers). Tooth surfaces were thoroughly cleaned with a cotton bud to remove polishing debris prior to indentation. The buccal enamel was examined for both incisors and molars. Microhardness measurements were carried out using a Duramin Microhardness Tester (Struers) using Duramin 5 software. The indentations were made using a Knoop diamond under a 100 g load for 15 s. A minimum of 10 indentations spaced at least 50 μm apart were made for each tooth region. Enamel 50–70 μm from the tooth surface or enamel 50–70 μm from the DEJ was examined for both control and KLK4 teeth. In addition, enamel from a region between these measurements was also examined in KLK4 teeth in order to compare with readings closer to the DEJ. The length of each indent was measured by image analysis and the microhardness reading was automatically calculated by the Duramin 5 software. Statistical testing used unpaired 2-tailed *T*-tests to compare results between regions within the same tooth. No tests were conducted to compare microhardness values between different teeth due to the small sample size.

### Energy-dispersive X-ray spectroscopy

Energy- dispersive X-ray spectroscopy (EDX) elemental analysis was performed on selected regions of the enamel of control and KLK4 teeth using a detector fitted with an ultrathin window using Bruker Quantax Espirit software version 1.9.4 (Bruker). A minimum of five measurements were obtained for each specified enamel region. The mean composition of each enamel region was then calculated using atomic mass percentage measurements.

## Results

### Clinical presentation and genotyping

We identified five families living in the UK but originating from Pakistan, presenting with autosomal recessive AI in the absence of any clinically-obvious co-segregating disease. Each of the families exhibited a similar hypomaturation AI phenotype (Figure 1). None of the families were known to be related. A total of 16 individuals from the five families were recruited to the study for genotyping. WES was carried out using DNA from one individual from families 1, 2, 3 and 5. Depth of coverage indicated that mean coverage was 54.5, 57.7, 80.8 and 59.7 × for individuals from families 1, 2, 3, and 5 respectively (Supplementary Table 3). For families 1, 2, 3, and 5, an identical homozygous frameshift mutation in *KLK4*, c.632delT, p.(L211Rfs^*^37) (NM_004917.4), was identified in affected individuals after filtering of WES data. The mutation was confirmed by Sanger sequencing (Supplementary Figure 1) and segregated with the AI phenotype in all available family members except for family 1 IV:2. This individual self-reported as affected with AI but was never examined, and was heterozygous for the variant (Figure 1). SNP genotyping data for both IV:2 and V:1 confirmed that the two affected individuals did not share a homozygous region over *KLK4* (Supplementary Tables 4–6). Sequencing of the remaining coding exons of *KLK4* for IV:2 did not identify any additional variants. The cause of AI in family 1, IV:2 therefore remains unknown.

Due to the similar phenotype, mode of inheritance and ethnicity of individual V:3 of family 4, Sanger sequencing was used to check for the presence of an identical mutation in *KLK4*. This revealed that family 4 also carried the *KLK4* c.632delT variant that segregated with disease for all family members for which DNA was available. Due to the presence of the same c.632delT variant in five families originating from Pakistan, microsatellite markers flanking the variant were genotyped for four of these families to check the surrounding haplotype. Families 1 and 4 shared an identical haplotype surrounding the *KLK4* c.632delT variant over a genetic distance of at least 13.4 cM (approximately 7.27 Mb in this case; Supplementary Tables 2, 7). Families 2 and 3 did not share an identical haplotype flanking the *KLK4* variant.

Interrogation of publically available databases of variation, such as dbSNP147, ExAC v0.3.1 and Exome Variant Server, found that the variant was present in all three databases, although at very low frequencies and always as a heterozygous variant (Supplementary Table 8). The *KLK4* variant was assigned as variant ID #0000000189 in the University of Leeds AI LOVD (http://dna2.leeds.ac.uk/LOVD/) and was submitted to ClinVar, accession SCV000494588.

### Enamel phenotyping

Deciduous teeth were made available from male V:1 (family 1) through either natural exfoliation or clinical extraction (hereafter referred to as KLK4 teeth; Supplementary Figure 2). In total five deciduous KLK4 teeth and five deciduous and type matched control teeth were examined. The control teeth were obtained from an anonymised tissue bank therefore the teeth could not be age or sex matched. Two whole KLK4 teeth (a canine and a molar) and two type matched control teeth were analyzed by high-resolution CT. SEM and EDX analysis were carried out on one KLK4 incisor tooth and a type matched control tooth. Mineral density measurements were also made from these slices of incisor teeth after high-resolution CT analysis. Two KLK4 teeth (an incisor and a molar) and two type matched control teeth were subjected to microhardness testing.

**Figure 2:**
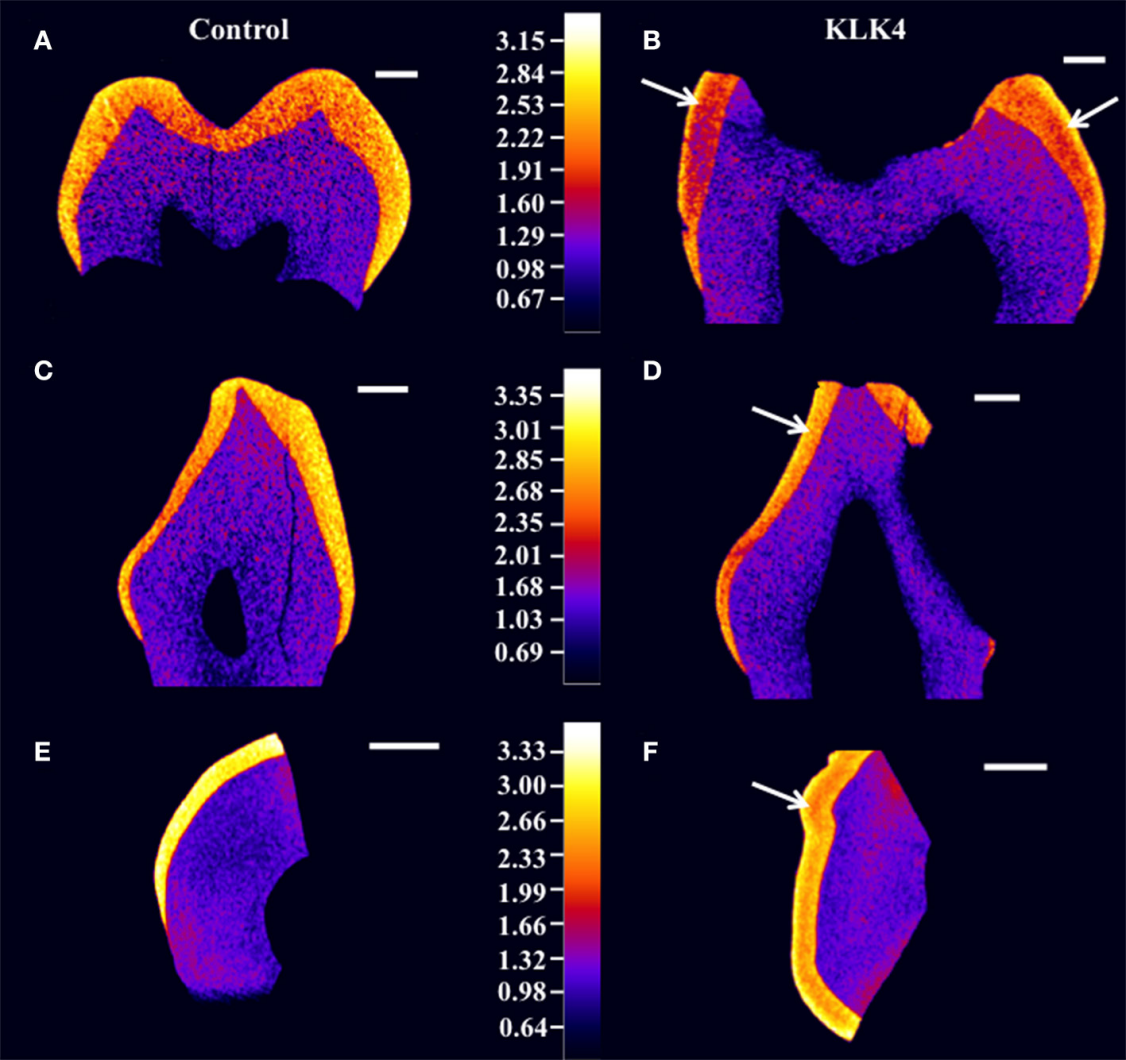
**High resolution X-ray CT analysis of WT control and KLK4 teeth from individual V:1 (family 1)**. **(A,B)**: molar teeth, **(C,D)**: canine teeth, **(E,F)**: incisal tooth slices. Arrows indicate the presence of an inner layer of enamel of lower mineral density not seen in control teeth. Scale bars represent 1 mm.

Structural analysis of the enamel was undertaken to characterize the detailed phenotype. High-resolution CT scans were carried out for two whole KLK4 teeth, including one canine and one molar, as well a 500 μm slice taken from a KLK4 incisor tooth (to reduce beam hardening), alongside dentition and type matched controls. CT scanning revealed that the enamel of the teeth from the *KLK4* c.632delT patient exhibited two distinct layers of different enamel density (Figure 2 and Supplementary Videos 1–4). For all tooth types, the outer layer extended a small distance from the surface and was consistently present except in those regions affected by, or lost as a result of, decay. This layer was most clearly visible in the regions of thicker enamel found at the enamel cusps and was not evident in control teeth.

Enamel mineral density measurements were taken from the 500 μm KLK4 and control tooth slices rather than from the whole teeth, since measurements from the tooth slices would be less affected by CT beam hardening artifacts. These errors could have led to artificial inflation of the outer enamel mineral density compared to the inner enamel mineral density. Therefore, to quantify the enamel mineral density in the outer and inner layers of the KLK4 tooth slice, specific regions were selected by application of mineral density thresholds. In this way, the entire enamel thickness and none of the dentine could be specifically selected by thresholding both samples at ≥2.13 g/cm^3^ (Supplementary Figure 3, Supplementary Table 9). For control enamel, the mean mineral density was 2.93 g/cm^3^, whereas the mean enamel mineral density for the KLK4 enamel was 2.64 g/cm^3^. By thresholding at a mineral density of ≥2.75 g/cm^3^, only the outer enamel layer in the KLK4 tooth slice was selected. However, the entire enamel thickness was selected in the control tooth slice, suggesting that the inner enamel layer seen in the KLK4 teeth was of reduced mineral density compared with both KLK4 outer enamel and the full width of enamel in control teeth. The mean enamel mineral values obtained for the control and the KLK4 tooth slice were 3.04 and 2.92 g/cm^3^ respectively. By thresholding at a mineral density of 2.13–2.75 g/cm^3^, only the inner enamel was selected in the KLK4 tooth slice and a mean value of 2.58 g/cm^3^ was obtained. No comparable data was obtainable for the control tooth slice as so little of the enamel had a mineral density value in this lower range. Whilst the enamel density of the control tooth showed a general trend of decreasing mineral density when moving from the outer enamel surface toward the DEJ, there were no clear divisions to demarcate sudden changes in enamel density, such as those seen in the KLK4 teeth (Figure 2).

**Figure 3:**
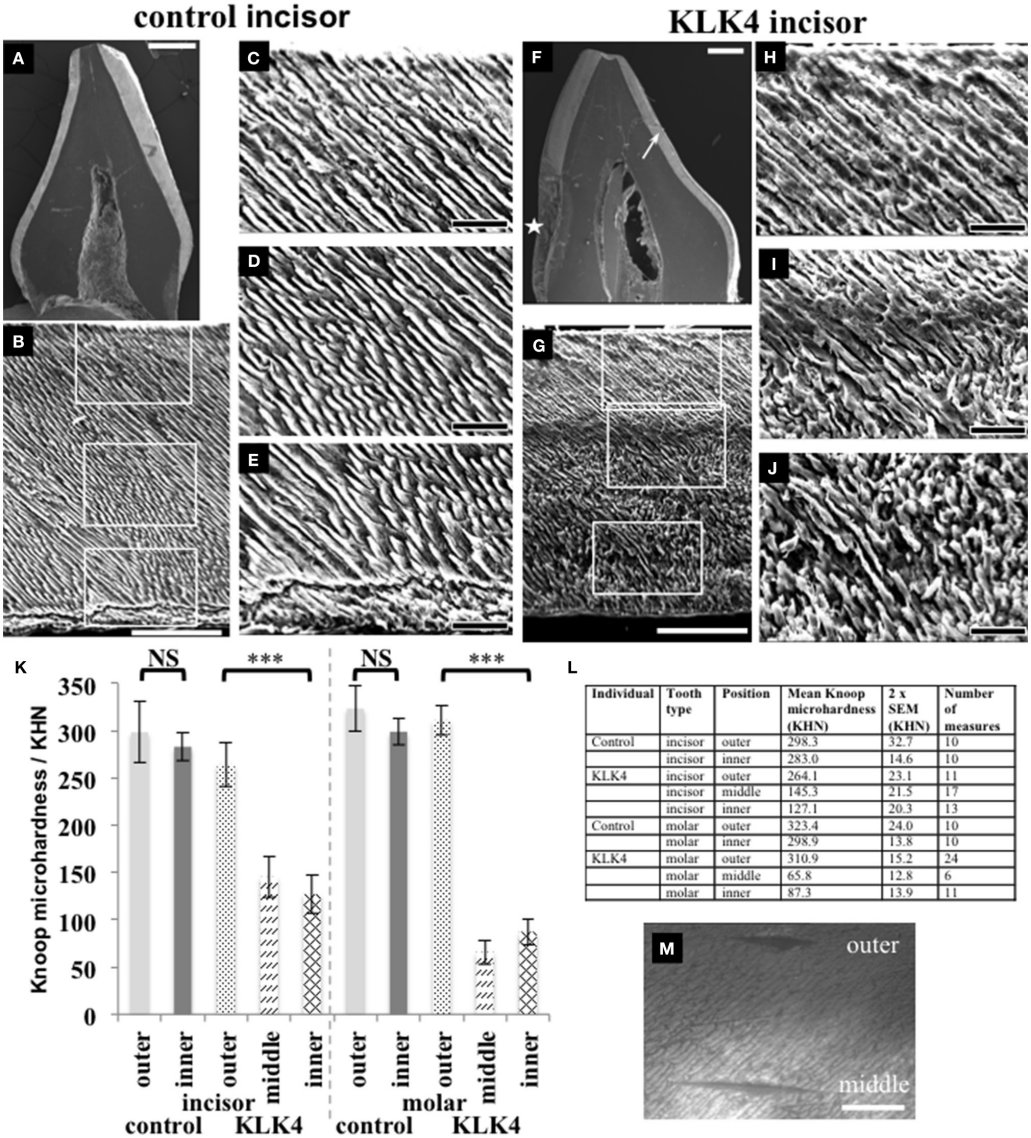
**SEM and microhardness testing of control and KLK4 teeth from individual V:1 (family 1)**. **(A–E)**: Control tooth; **(F–J)**: KLK4 tooth. **(A)**: Whole tooth longitudinal section. **(B)**: The enamel layer with the enamel surface shown at the top and the enamel-dentine junction at the bottom. White boxes indicate the positions of magnified images **(C,D)** and **(E)** respectively (top to bottom). **(C–E)**: outer **(C)**: close to the surface; middle **(D)** and inner **(E)**: close to the EDJ, enamel. **(F)**: Whole tooth longitudinal section—note the clear demarcation in the enamel layer between the inner and outer enamel on the lingual side of the tooth (arrow). An area of decay is present on the one side of the tooth (star). **(G)**: The enamel layer with surface at the top and the enamel-dentine junction at the bottom. White boxes indicate the positions of magnified images **(H–J)** respectively (top to bottom). **(H–J)**: outer **(H)**: close to the surface: middle **(I)** and inner **(J)**: close to the EDJ, enamel. **(K)**: Knoop microhardness testing for control and KLK4 incisor and molar teeth. Error bars represent 2x standard error of the mean. Brackets indicate the conditions for which unpaired, two-tailed *T*-tests were undertaken. Results are abbreviated as follows: NS; not significant, i.e., *p* > 0.05; ^***^ significant at *p* < 0.001. **(L)**: Knoop microhardness testing results in table form. **(M)**: Light microscopy image showing two indentations made around 100 μm apart on the KLK4 incisor tooth, one in the outer enamel layer and the other in the middle enamel layer. Scale bars represent **(A,F)**: 1 mm; **(B,G)**: 100 μm; **(C–E)** and **(H–J)**: 25 μm; **(M)**: 50 μm.

For SEM, one KLK4 incisor tooth and one type matched control tooth were sectioned planar to the bucco-lingual axis. SEM revealed that a clear demarcation boundary between the outer and the inner enamel was visible for the KLK4 tooth, most especially on the lingual face, but no such boundary was evident for the control tooth (Figure 3). In addition, the outer enamel of the KLK4 incisor tooth had the appearance of a more densely packed mineral compared with the inner enamel of the KLK4 incisor tooth.

Knoop microhardness (KM) testing was carried out to determine whether the lower mineral density detected in the inner layer of KLK4 teeth resulted in lower KM values compared to control teeth. One KLK4 incisor tooth and one KLK4 molar tooth and two type matched controls were analyzed along the buccal faces of the enamel in each section. Measurements revealed that the outer enamel of control incisor and molar teeth had a mean KM value of 298.3 ± 32.7 (2x standard error of the mean) and 323.4 ± 24.0 respectively. Control inner enamel had mean KM values of 283.0 ± 14.6 (incisor) and 298.9 ± 13.8 (molar) (Figure 3). These data agree with previous investigations of control human deciduous teeth, showing that microhardness decreases from the surface enamel toward the DEJ (He et al., 2010) with reported KM values ranging from 304.5 to 326.6 for incisors (25 g load for 30 s, Scatena et al., 2014) and 271–320 for molars (50 g load for 10 s, Mirkarimi et al., 2012; Mudumba et al., 2014). For the KLK4 teeth, the KM of the outer enamel layer was 264.1 ± 23.1 for the incisor and 310.9 ± 15.2 for the molar, representing 88.5 and 96.1 % of the values obtained for matched control teeth respectively. KM values for the inner enamel of the KLK4 teeth however, were much reduced, at 127.1 ± 20.3 (incisor) and 87 ± 13.9 (molar) and were significantly lower (incisor: *t* = −8.9486 *p* < 0.001; molar: *t* = −18.2654, *p* < 0.001) than the values obtained for the KLK4 outer enamel (Figure 3M). Comparison of the KM values for the inner and outer enamel of both the matched control incisor and molar teeth showed that the KM of the inner enamel was not significantly different to that of the outer enamel in each case (incisor: *t* = −0.854 *p* > 0.05; molar: *t* = −1.7642, *p* > 0.05).

**Figure 4:**
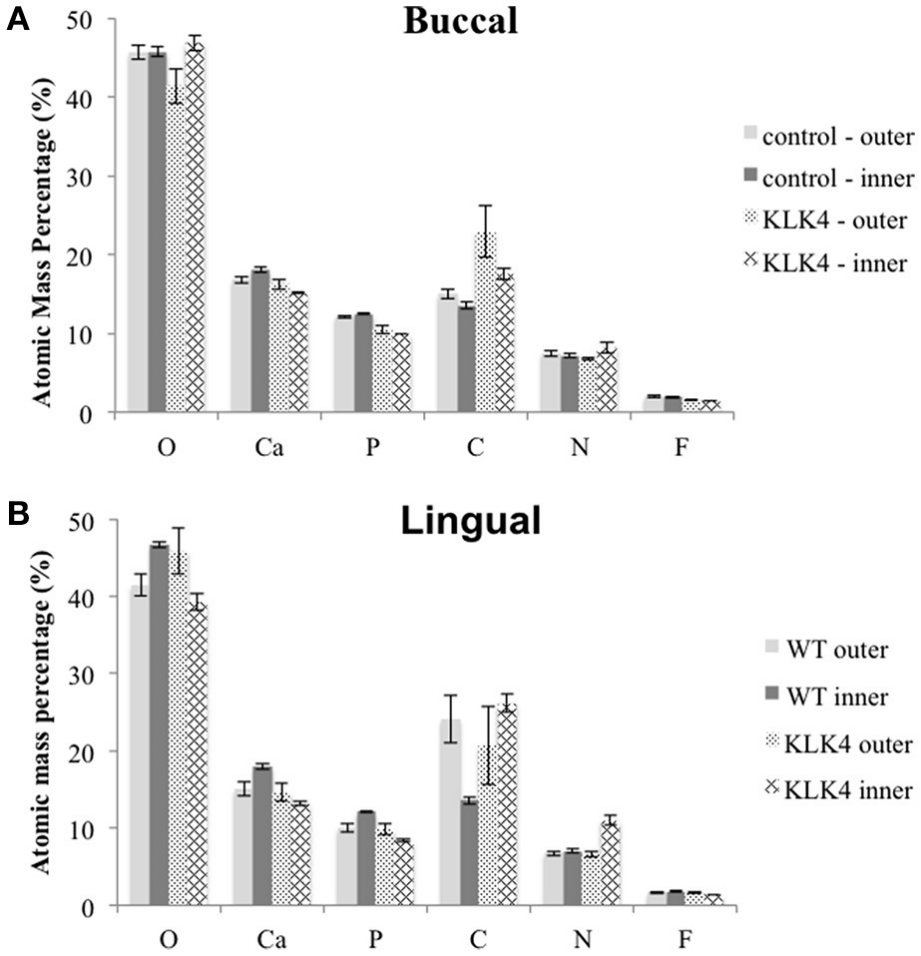
**Elemental analysis by energy dispersive X-ray spectroscopy (EDX)**. Measurements are detailed in Supplementary Table 10. Note that Mg and Na content were also analyzed but are not included here. Measurements were taken from both sides of the tooth: **(A)**: buccal, **(B)**: lingual.

EDX analysis was undertaken on the lingual and buccal outer and inner enamel for the KLK4 incisor tooth and the type matched control tooth previously analyzed using SEM. Compared to the control tooth, the inner enamel of the KLK4 tooth contained a lower atomic percentage of both calcium and phosphorus (Figure 4, Supplementary Table 10). KLK4 inner enamel also contained a larger atomic percentage proportion of nitrogen although this was more evident for the enamel on the lingual side of the tooth than the buccal side. The atomic percentage content of both oxygen and carbon appeared to vary across both teeth while the atomic percentage contribution of fluoride, sodium and magnesium were all found to be lower in the KLK4 tooth compared with the control tooth.

## Discussion

The *KLK4* variant, identified here in five unrelated families of Pakistani origin, was associated with a hypomaturation AI clinical phenotype, as previously described for the three other *KLK4* variants reported so far (Hart et al., 2004; Wright et al., 2011; Wang et al., 2013; Seymen et al., 2015). The *KLK4* c.632delT variant is predicted to produce a transcript that escapes NMD as it is predicted to lead to a frameshift variant in the final exon. RNA was unavailable from any of the families for study to confirm this. Of the *KLK4* variants previously identified, only the c.620_621delCT, p.S207Wfs^*^38 variant leads to a frameshift, generating a premature stop codon within the final exon (Seymen et al., 2015), whereas the other two reported mutations (a frameshift and a nonsense variant) are predicted to produce a transcript that is subject to NMD (Hart et al., 2004; Wang et al., 2013). Seymen et al. (2015) analyzed the effect of the *KLK4* c.620_621delCT, p.(S207Wfs^*^38) variant via an *in vitro* expression assay and found reduced protein expression compared with a WT construct and no catalytic activity. This was unsurprising since the frameshift affected the p.S207 residue, one of the catalytic triad of residues crucial to the function of all kallikrein enzymes.

A truncated protein may potentially be produced as a result of the *KLK4* c.632delT variant reported here. If this were indeed the case, the catalytic triad of residues essential to the function of KLK4 (residues His71, Asp116, and Ser207) would be preserved but three of six structurally important disulphide bonds would be precluded due to loss of cysteine residues at positions 213, 228, and 241 (Debela et al., 2006) (Supplementary Figure 4). The protein is therefore likely to be misfolded and may be degraded or non-functional.

The recognition of an identical *KLK4* variant within five families not known to be related but of the same Pakistani origin, suggests that the mutation may be the result of a common ancestral founder mutation. Analysis of the haplotype that surrounded the *KLK4* c.632delT variant in four of the families (1–4) found that families 1 and 4 shared a relatively large common haplotype extending at least 13.4 cM (approximately 7.27 Mb), but there was no evidence of a shared haplotype for families 2 and 3. This, together with the relative frequency of the variant within the South Asian population (0.146 %) and the lack of the variant in other populations (other than in one individual of African origin) as shown by the ExAC browser (v0.3.1), all suggest that the c.632delT variant may have arisen in the Pakistani population a long time ago and may have been maintained in several isolated endogamous sub-populations. Overall, these findings suggest that the *KLK4* c.632delT variant may be a common cause of autosomal recessive AI in the Pakistani population.

Phenotypic analysis of teeth from individual V:1 from family 1 using CT showed that the enamel was hypomineralised in comparison to control enamel but that the hypomineralisation was greatest in the deeper enamel, away from the surface and toward the DEJ. Microhardness readings also showed that the surface enamel was harder than the inner enamel layer and a clearly demarcated outer enamel layer was visible upon SEM. EDX revealed decreased oxygen and phosphorus content, and hinted at increased nitrogen content for the inner enamel of the KLK4 tooth compared with the WT control. This latter may suggest the presence of retained protein but this remains to be confirmed.

This apparent difference in the mineralization, elemental content and hardness of KLK4 inner compared with outer enamel may reflect a number of different factors acting during enamel formation.

During the transition and maturation stages, ameloblasts secrete KLK4 into the enamel matrix to further degrade enamel matrix proteins already processed by the secretory stage proteinase, MMP20. *KLK4* has been shown to be a relatively recent evolutionary development that is suggested to be concurrent with the development of thicker enamel and earlier tooth eruption (Kawasaki et al., 2014). Prior to *KLK4* evolution, ameloblast endocytosis of enamel matrix protein fragments was the primary mechanism for elimination of residual protein from the maturing tissue, though residual MMP20 secreted during the secretory stage may have also remained active to cleave enamel peptides. It has been shown *in vitro* that MMP20 is able to activate KLK4 and is itself inactivated by the action of KLK4, thus potentially explaining the shift in proteinase activity from MMP20 to KLK4 observed during the transition stage of enamel development (Yamakoshi et al., 2013). If mutant KLK4 were unable to inactivate MMP20, MMP20 may remain active during the maturation stage as reported in *Klk4*^−/−^ mice (Yamakoshi et al., 2011). However this alone would not explain the greater mineralization of the outer enamel layer compared to the inner enamel in the KLK4 teeth. It has been suggested that cleavage by KLK4 enables the movement of enamel matrix protein degradation products from the deeper enamel (Bartlett and Simmer, 2014) and an absence or reduced activity of KLK4 would preclude or hinder this. In such a case, residual protein fragments would remain in the inner depths of the tissue, inhibiting secondary mineral growth of the enamel crystallites and leading to reduced mineral density. *Klk4*^−/−^ mouse studies support this theory as the enamel has been shown to be less mineralized and softer with depth whilst the overall enamel thickness is similar to that of WT mice (Simmer et al., 2011; Smith et al., 2011; Nunez et al., 2016).

The mean enamel mineral density reported for *Klk4*^−/−^ mice was 2.5 g/cm^3^ compared with 3.1 g/cm^3^ reported for WT controls, a reduction of 19 % (Nunez et al., 2016). The figures reported here for enamel density measurements in the human *KLK4* c.632delT tooth are equivalent to a 10% reduction, although it is important to stress that this is based on measurements at only one plane in one tooth slice. Microhardness readings for *Klk4*^−/−^ mice indicated a 77 and 39 % reduction in microhardness between the inner and outer enamel respectively (Nunez et al., 2016). This compares with 55–71% and 4–11% reductions in microhardness between the inner and outer enamel of our human *KLK4* c.632delT teeth vs. matched control teeth. It must be noted that the loads used, and the time for which they were applied during microhardness testing may account for some of the differences in absolute values obtained in mouse compared with human teeth. However, there are a number of other differences in amelogenesis between the two species, including the speed of enamel formation, the thickness of the enamel formed, the architecture of the two tissues and the fact that enamel formed in the null mouse model may not be comparable to enamel from an individual with a *KLK4* mutation predicted to escape NMD. Nevertheless, our data clearly demonstrate a difference in hardness between inner and outer enamel of KLK4 teeth that is not apparent in human control teeth and is similar to that seen in the *Klk4*^−/−^ mouse, suggesting that the inner enamel is preferentially affected by perturbations in KLK4 function.

During maturation, ameloblasts employ endocytosis to remove residual enamel matrix proteins/peptides diffusing to the surface/outer enamel layer from deeper enamel layers. Reduced proteolysis due to compromised KLK4 activity may hinder diffusion from deeper layers and may result in retention of proteins/peptide within the deeper enamel. This may, in turn, inhibit maturation stage crystal growth. The visible demarcation between the outer and inner enamel in our KLK4 teeth and the observed differences in physical properties between inner and outer enamel in both the *Klk4*^−/−^mouse model and the KLK4 human teeth could represent the extent to which ameloblast endocytosis alone is effective in removing residual matrix protein. Given the more rapid time to eruption in the mouse compared with the human, this might also explain the differences in absolute hardness values between our KLK4 human enamel and the *Klk4*^−/−^ mouse teeth, with the longer maturation time in the case of human enamel development resulting in (relatively) harder tissue. It is difficult to explain the developmental origin of the clear demarcation line present between the outer and inner enamel, but it may actually be a preparation artifact reflecting some subtle difference in the physiochemical properties of the enamel involved.

In addition, post-eruptive mineralization of surface enamel by ions from saliva may have contributed to the outer enamel being harder than the inner enamel of the KLK4 teeth. The extent to which post-eruptive mineralization can compensate for hypomineralisation in the outer enamel of human teeth is unknown but cannot be discounted.

In conclusion, the data presented here adds a fourth *KLK4* mutation to those already reported and challenges the notion that *KLK4* mutations are a rare cause of AI, at least in the Pakistani population. The c.632delT variant may represent a founder mutation within the Pakistani population and may prove to be a common cause of autosomal recessive AI in this cohort. Analysis of human enamel affected by the *KLK4* c.632delT mutation has shown common features with those described for enamel in the *Klk4*^−/−^ mouse model, suggesting that KLK4 is essential for protein removal from, and the mineralization of, the inner enamel matrix.

## Ethics statement

This study was carried out in accordance with the recommendations of the principles outlined in the declaration of Helsinki and with local ethical approval from the National Research Ethics Service Committee, UK with written informed consent from all subjects. All subjects gave written informed consent in accordance with the Declaration of Helsinki. The protocol was approved by the National Research Ethics Service Committee, UK: Yorkshire and The Humber-South Yorkshire Research Ethics Committee reference 13/YH/0028.

## Author contributions

CS contributed to study design, conducted all experimental work, except that stated below, and drafted the manuscript. JK contributed to study design and edited the manuscript. CI and AM contributed to study design. PD, FS, EM, and AM recruited the families. JP contributed to study design, carried out SNP genotyping analysis, and prepared WES libraries for family 1. SB contributed to study design and advised on enamel phenotyping. All authors read and critically revised the manuscript. All authors gave final approval and agree to be accountable for all aspects of the work.

### Conflict of interest statement

The authors declare that the research was conducted in the absence of any commercial or financial relationships that could be construed as a potential conflict of interest.

## Supplementary material

Supplementary Video 1 **Control canine**.

Supplementary Video 2 **KLK4 canine**.

Supplementary Video 3 **Control molar**.

Supplementary Video 4 **KLK4 molar**.
