# Supplementary material for: A Fourth KLK4 Mutation Is Associated with Enamel Hypomineralisation and Structural Abnormalities
Source: Front Physiol. 2017 May 29;8:333. doi: 10.3389/fphys.2017.00333 (PMC5447068; doi:10.3389/fphys.2017.00333)
Supplement: Supplementary file 5 [file DataSheet1.DOCX]

Supplementary Material

**A Novel *KLK4* Mutation Is Associated With Enamel Hypomineralisation And Structural Abnormalities**

**Claire E.L. Smith^*^, Jennifer Kirkham, Peter F. Day, Esther J. McDerra, James A. Poulter, Christopher F. Inglehearn, Alan J. Mighell and Steven J. Brookes.**

*** Correspondence:** Dr Claire E. L. Smith: c.e.l.smith@leeds.ac.uk


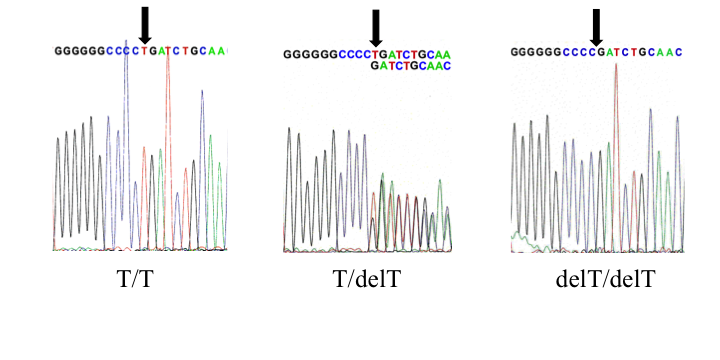


**Supplementary Figure 1.** Sanger sequencing of the *KLK4* c.632delT variant. Arrows indicate the position of the c.632 nucleotide (NM_004917.4).


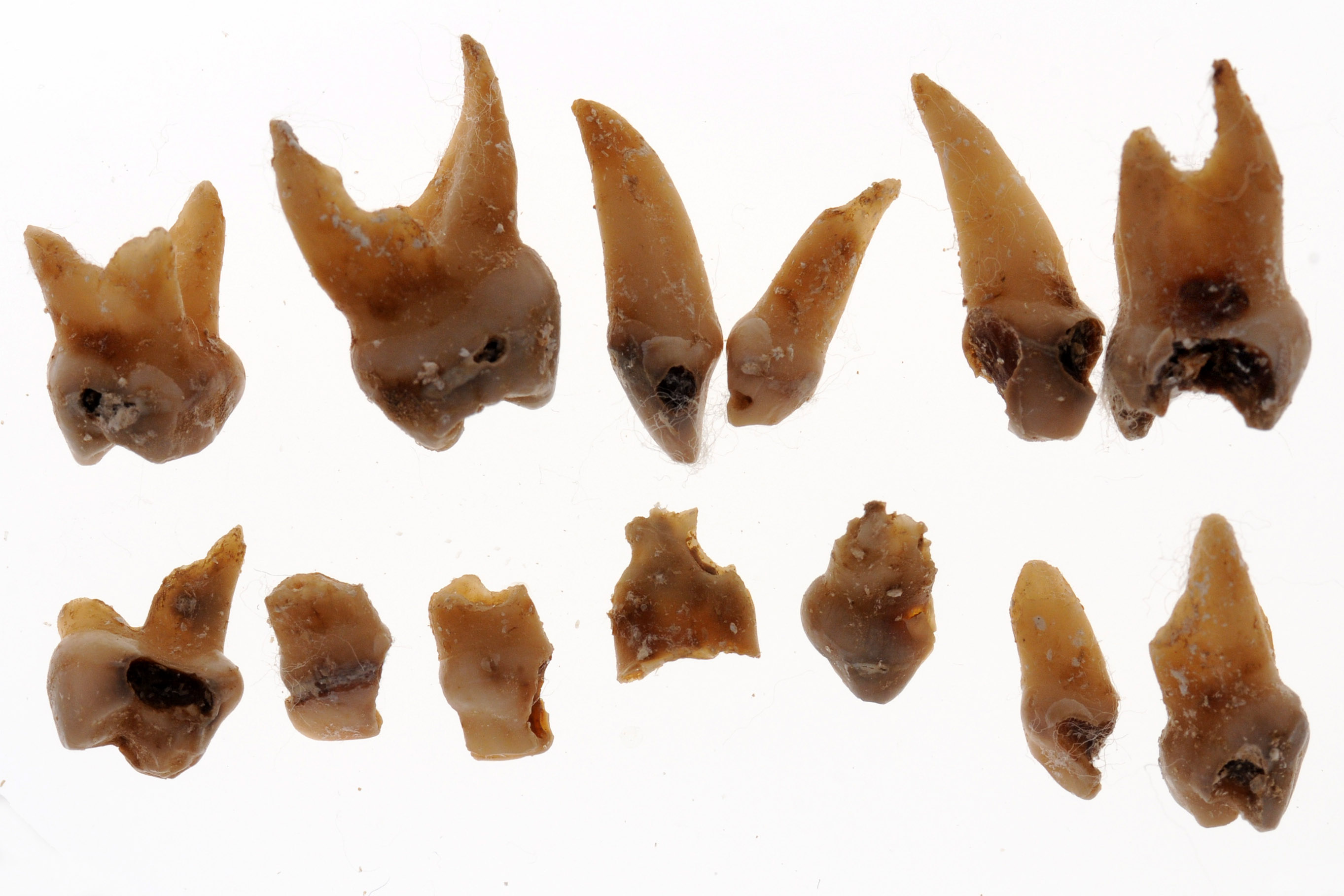


**Supplementary Figure 2.** Teeth obtained from family 1, V:1.

**
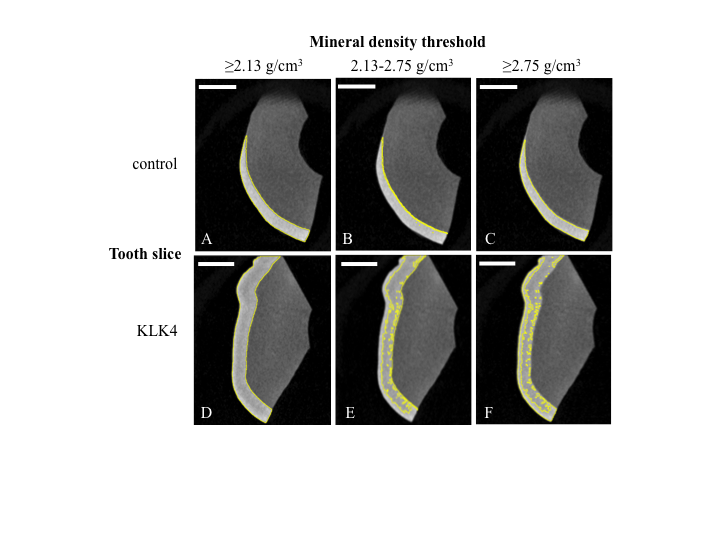
Supplementary Figure 3.** Calibrated, mineral density thresholded, high resolution X-ray CT scans of 500μm tooth slices. Tooth slices of 500μm thickness were scanned to reduce the effect of beam hardening on mineral density measurements, (A)-(C): control tooth slice, (D)-(F): KLK4 tooth slice. Enamel mineral density measurements were taken for enamel regions selected by application of calibrated mineral density thresholds to the grey-scale image. Regions enclosed by yellow lines are those selected by the threshold. Grey-scale artefacts at the outer edge of each sample were removed. Note that for the control tooth slice, thresholding of 2.13-2.75 g/cm^3^ selects only the enamel-dentine junction (B), whereas for KLK4 tooth slice (E), the majority of the inner enamel layer is selected. (A) and (D): entire enamel layer selected by threshold of ≥2.13 g/cm^3^_._ (B) and (E): selection of enamel with mineral density of 2.13-2.75 g/cm^3^. **(**C) and (F): selection of enamel with mineral density of ≥2.75 g/cm^3^. Mean measurements of enamel mineral density for the selected regions are detailed in Supplementary Table 9.

**
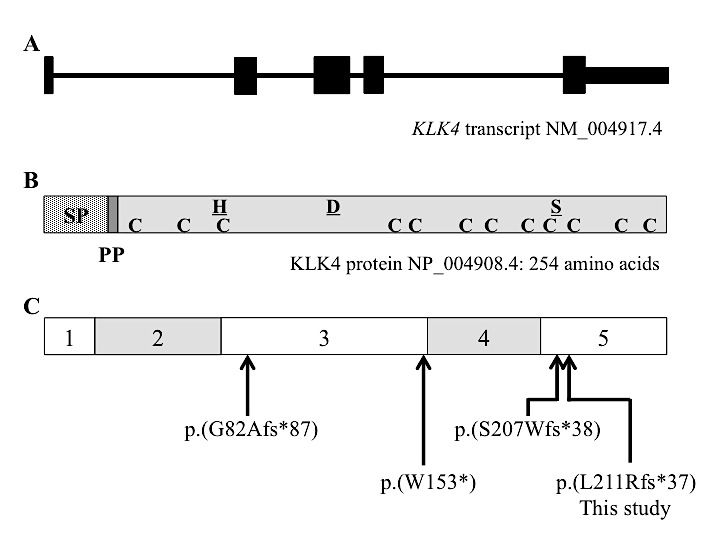
Supplementary Figure 4.** *KLK4* transcript, KLK4 protein structure and mutations reported in patients with AI. (A): The *KLK4* transcript (NM_004917.4) contains 5 exons. (B): The KLK4 protein (NP_004908.4) includes an N terminal signal peptide and propeptide, 6 disulphide bridges formed by 12 Cys (C) residues and a catalytic triad of His (H), Asp (D), Ser (S) residues. **(**C): The position of the residues impacted by the *KLK4* mutations identified in this and in previous studies are shown relative to the position in the exon where they are encoded. Numbers indicate the parts of the protein encoded by each exon (NM_004917.4). Abbreviations: PP propeptide; SP signal peptide.

| **Amplicon** | **Forward sequence** | **Reverse sequence** | **Product size (bp)** |
| --- | --- | --- | --- |
| *KLK4* exon 5 | TCTGGAATGGGACTTCCAAC | AGGGGCTGGGAACAGATATT | 391 |

**Supplementary Table 1.** Primer sequence used to verify the *KLK4* c.632delT variant (NM_004917.4) by Sanger sequencing.

| **Marker** | **Physical location (chr19: hg19/b37)** | **Genetic location (Marshfield; cM)** | **Forward primer** | **Reverse primer** | **Size (bp)** |
| --- | --- | --- | --- | --- | --- |
| D19S217 | 44281363-44281698 | 68.08 | GGGGTGGGATTGAAGTTGGTT | CAAGACCCATACCCATGA | 219-233 |
| D19S902 | 48332028-48332279 | 72.72 | CCATCCTAATGAGGGCAA | GCACCAGTGACTGCCTGT | 199-217 |
| D19S246 | 50955585-50955921 | 78.08 | AGAGTGAGATTCCACCTTTC | GAAACACATCATTTACCCAC | 185-229 |
| D19S907 | 51061251-51061560 | 78.08 | GTGTCCAATCAACAGACCA | CTGCACTCCAGCAGAAAT | 213-223 |
| *KLK4* c.632delT | 51410323 | N/A | N/A | N/A | N/A |
| D19S553 | 51549504- 51550032 | 81.51 | CATGCCTCTAGTCCCAGCT | GACAAATGCCAGAAAGCCTG | 362-479 |
| D19S397 | 52453100-52453665 | 82.18 | CATTGTACTCCAGCCTGGG | CTCACAGTTTTCACAATTGCA | 245 |
| D19S601 | 52593833-5294230 | 83.19 | CAATGTGAGGCTGGTCTCTT | ATCATATGGCCTTCAGTGGA | 137-228 |

**Supplementary Table 2.** Microsatellite markers used to genotype the region surrounding the *KLK4* c.632delT variant (NM_004917.4).

| **Sample (Family)** | **Mean coverage** | **% of bases covered by** | | | | | |
| --- | --- | --- | --- | --- | --- | --- | --- |
|  |  | **≥5x** | **≥10x** | **≥15x** | **≥20x** | **≥25x** | **≥30x** |
| V:1 (Family 1) | 54.53 | 94.9 | 93.0 | 90.0 | 85.6 | 80.1 | 73.9 |
| I:2 (Family 2) | 57.72 | 98.7 | 97.2 | 94.5 | 90.3 | 84.9 | 78.7 |
| III:2 (Family 3) | 80.77 | 99.0 | 97.9 | 96.4 | 94.1 | 91.1 | 87.4 |
| II:2 (Family 5) | 59.65 | 99.0 | 96.9 | 93.5 | 88.8 | 83.1 | 76.7 |

**Supplementary Table 3.** WES depth of coverage statistics. The regions captured by the Agilent SureSelect version 5 (families 1, 2 and 3) or version 6 (family 5) reagent were used as the interval. NB DNA from unaffected but obligate heterozygous carrier I:2 underwent WES for family 2.

| **Region (hg19/b37)** | **Flanking SNPs** | **Size (bp)** |
| --- | --- | --- |
| chr1:32146764-33221282 | rs2297684;rs360490 | 1,074,518 bp |
| chr1:35416760-36551895 | rs12568660;rs408986 | 1,135,135 bp |
| chr1:149040317-150506263 | rs11588570;rs11586047 | 1,465,946 bp |
| chr1:152613472-156224304 | rs11205056;rs7542798 | 3,610,832 bp |
| chr2:199352368-200643494 | rs4413156;rs7578914 | 1,291,126 bp |
| chr2:210377109-211465780 | rs17317114;rs2371000 | 1,088,671 bp |
| chr3:24746320-31857836 | rs4413265;rs13087711 | 7,111,516 bp |
| chr3:48152659-49213030 | rs4858883;rs11713297 | 1,060,371 bp |
| chr3:49902160-52268866 | rs2883059;rs353547 | 2,366,706 bp |
| chr3:88788675-93638017 | rs1483476;rs7428007 | 4,849,342 bp |
| chr3:109503214-117243036 | rs688405;rs16827097 | 7,739,822 bp |
| chr4:44578736-45638847 | rs16857127;rs2061231 | 1,060,111 bp |
| chr5:92627491-93896128 | rs11135569;rs6898585 | 1,268,637 bp |
| chr6:114969280-116006694 | rs12190018;rs6912763 | 1,037,414 bp |
| chr6:121902869-123104205 | rs4541776;rs9490549 | 1,201,336 bp |
| chr7:68718888-69865264 | rs10231898;rs10950200 | 1,146,376 bp |
| chr7:86983715-88019109 | rs7808249;rs11773152 | 1,035,394 bp |
| chr7:117697862-120731577 | rs1859029;rs6466767 | 3,033,715 bp |
| chr8:99546681-100994389 | rs4332102;rs6468704 | 1,447,708 bp |
| chr9:27453327-36901494 | rs10812599;rs17391944 | 9,448,167 bp |
| chr9:123127308-124281184 | rs10760095;rs7029360 | 1,153,876 bp |
| chr10:21567724-22791686 | rs3895938;rs12770182 | 1,223,962 bp |
| chr10:34686067-36003561 | rs3844127;rs687812 | 1,317,494 bp |
| chr10:115372585-119606119 | rs17090764;rs11198145 | 4,233,534 bp |
| chr10:127731365-129140763 | rs7919338;rs2386830 | 1,409,398 bp |
| chr11:1-11333188 | rs3802985;rs4909985 | 11,333,187 bp |
| chr11:51441936-55715697 | rs560488;rs17513829 | 4,273,761 bp |
| chr12:102012882-103101412 | rs825090;rs1463770 | 1,088,530 bp |
| chr13:44627157-47342773 | rs17462790;rs1216987 | 2,715,616 bp |
| chr13:87104192-90464520 | rs1566659;rs1932302 | 3,360,328 bp |
| chr14:105971894-107349540 | rs6576110;rs17112143 | 1,377,646 bp |
| chr15:28239735-29381801 | rs2077596;rs12442802 | 1,142,066 bp |
| chr15:50497848-52860553 | rs2278167;rs1724611 | 2,362,705 bp |
| chr15:71967546-73246440 | rs931728;rs11072398 | 1,278,894 bp |
| chr16:14151672-16858308 | rs2011209;rs9936999 | 2,706,636 bp |
| chr16:46723598-48202503 | rs33994299;rs17821664 | 1,478,905 bp |
| chr16:58327774-65546332 | rs2288014;rs4785833 | 7,218,558 bp |
| chr19:36666167-38505645 | rs2112971;rs7258557 | 1,839,478 bp |
| chr20:34191629-35272778 | rs6060499;rs221310 | 1,081,149 bp |
| chr21:29803555-31140753 | rs2250911;rs459617 | 1,337,198 bp |
| chr21:38077544-44754437 | rs11909356;rs586330 | 6,676,893 bp |
| chr22:41071659-42717108 | rs133063;rs1107526 | 1,645,449 bp |

**Supplementary Table 4.** Homozygous regions identified by SNP genotyping for family 1, IV:2. Only regions of ≥1 Mb, containing ≥25 SNPs are included.

| **Region (hg19/b37)** | **Flanking SNPs** | **Size (bp)** |
| --- | --- | --- |
| chr1:35636227-36909385 | rs11264110;rs10908303 | 1,273,158 bp |
| chr1:53590630-79238015 | rs3766793;rs4650608 | 25,647,385 bp |
| chr1:96316702-97575076 | rs4950088;rs12129287 | 1,258,374 bp |
| chr1:102744309-104572622 | rs10874571;rs12031508 | 1,828,313 bp |
| chr1:107931160-109727284 | rs746603;rs648673 | 1,796,124 bp |
| chr1:155197462-156213257 | rs2990245;rs759330 | 1,015,795 bp |
| chr1:173542864-175090414 | rs6691482;rs6703913 | 1,547,550 bp |
| chr2:88759965-104755310 | rs11693918;rs7588406 | 15,995,345 bp |
| chr2:134359385-157564814 | rs11904377;rs1564083 | 23,205,429 bp |
| chr2:222847214-225828821 | rs16863257;rs10210032 | 2,981,607 bp |
| chr3:13140835-17208267 | rs360749;rs1138454 | 4,067,432 bp |
| chr3:49902160-51874679 | rs2883059;rs16943 | 1,972,519 bp |
| chr3:157536183-158537110 | rs16828352;rs2615055 | 1,000,927 bp |
| chr4:8686173-9915741 | rs7378503;rs11722228 | 1,229,568 bp |
| chr4:43277772-44292501 | rs7689923;rs1602290 | 1,014,729 bp |
| chr5:86683105-99049448 | rs1062035;rs10046017 | 12,366,343 bp |
| chr5:129885155-131327157 | rs7731850;rs421377 | 1,442,002 bp |
| chr6:31098957-32685865 | rs13200022;rs9275653 | 1,586,908 bp |
| chr6:55254659-56341981 | rs9475279;rs2273973 | 1,087,322 bp |
| chr6:91086462-137095679 | rs790595;rs9321570 | 46,009,217 bp |
| chr7:5608932-16014635 | rs852499;rs7787656 | 10,405,703 bp |
| chr7:67946524-70016901 | rs1554496;rs4718959 | 2,070,377 bp |
| chr7:126732811-134310112 | rs17866036;rs1160049 | 7,577,301 bp |
| chr7:141612621-144647694 | rs11767947;rs35029981 | 3,035,073 bp |
| chr8:14161143-16988955 | rs17119030;rs10093037 | 2,827,812 bp |
| chr8:50293544-51966664 | rs7839023;rs7845197 | 1,673,120 bp |
| chr8:85108639-86217265 | rs7822580;rs13257749 | 1,108,626 bp |
| chr8:97221918-98231744 | rs10104165;rs11784000 | 1,009,826 bp |
| chr8:142882029-146364022 | rs10110903;rs6991681 | 3,481,993 bp |
| chr9:8283179-13508282 | rs1500307;rs3737150 | 5,225,103 bp |
| chr9:90195972-120265254 | rs11141903;rs524222 | 30,069,282 bp |
| chr9:138428645-141213431 | rs210081;rs4295734 | 2,784,786 bp |
| chr10:6064181-13762810 | rs11596355;rs11258556 | 7,698,629 bp |
| chr10:100280492-101334234 | rs11594030;rs12414093 | 1,053,742 bp |
| chr11:38145657-39282575 | rs10836933;rs10837134 | 1,136,918 bp |
| chr11:89962734-128190758 | rs2155056;rs7935417 | 38,228,024 bp |
| chr12:34363279-38843441 | rs10844839;rs7961495 | 4,480,162 bp |
| chr13:19238083-20798240 | rs4037485;rs877098 | 1,560,157 bp |
| chr13:24680274-28452129 | rs9551060;rs1778797 | 3,771,855 bp |
| chr13:87104192-90464520 | rs1566659;rs1932302 | 3,360,328 bp |
| chr14:66506241-67937892 | rs1273870;rs1315732 | 1,431,651 bp |
| chr15:23222284-25732610 | rs11263693;rs4906716 | 2,510,326 bp |
| chr16:46723598-48202503 | rs33994299;rs17821664 | 1,478,905 bp |
| chr17:58154207-59296426 | rs12951337;rs17597484 | 1,142,219 bp |
| chr17:79191726-80394530 | rs9896850;rs4789777 | 1,202,804 bp |
| chr19:15689452-17861868 | rs10422455;rs12978286 | 2,172,416 bp |
| chr19:48476964-56637730 | rs2972521;rs902525 | 8,160,766 bp |
| chr20:26167980-30433139 | rs845779;rs6089096 | 4,265,159 bp |
| chr22:40708679-42259098 | rs2072858;rs714015 | 1,550,419 bp |
| chr22:44391025-46868281 | rs5764055;rs7292762 | 2,477,256 bp |

**Supplementary Table 5.** Homozygous regions identified by SNP genotyping for family 1, V:1. Only regions of ≥1 Mb, containing ≥25 SNPs are included.

| **Region (hg19/b37)** | **Flanking SNPs** | **Size (bp)** |
| --- | --- | --- |
| chr1:35636227-36551895 | rs11264110;rs408986 | 915,668 bp |
| chr1:155197462-156213257 | rs2990245;rs759330 | 1,015,795 bp |
| chr3:49902160-51874679 | rs2883059;rs16943 | 1,972,519 bp |
| chr13:87104192-90464520 | rs1566659;rs1932302 | 3,360,328 bp |
| chr16:46723598-48202503 | rs33994299;rs17821664 | 1,478,905 bp |
| chr22:41071659-42259098 | rs133063;rs714015 | 1,187,439 bp |

**Supplementary Table 6.** Homozygous regions shared by family 1, IV:2 and V:1 identified by SNP genotyping. Only overlapping regions of 10,000 Kb (≥1 Mb and containing ≥25 SNPs per sample) are included.

| **Family** | **Marker / Genetic Location (Marshfield / cM)** | | | | | | | |
| --- | --- | --- | --- | --- | --- | --- | --- | --- |
|  | D19S217 | D19S902 | D19S246 | D19S907 | c.632delT | D19S553 | D19S397 | D19S601 |
|  | 68.08 | 72.72 | 78.08 | 78.08 | - | 81.51 | 82.18 | 83.19 |
| 1 | 228 | 208 | 226 | 215 | -/- | 464 | NR | 208 |
| 2 | 224 | 212 | 182 | 213 | -/- | 442 | 256 | 212 |
| 3 | 224 | 212 | 226 | 219 | -/- | 460 | 256 | 208 |
| 4 | 228 | 208 | 226 | 215 | -/- | 464 | NR | NR |

**Supplementary Table 7.** Genotyping results for the region flanking the *KLK4* c.632delT variant (NM_004917.4) for families 1-4. The grey shaded region indicates the region of the shared haplotype for families 1 and 4. Genotyping was not carried out for family 5. Abbreviation: NR no result.

| **Gene** | **Mutation (NM_004917.4)** | **Position (GRCh37/hg19)** | **dbSNP147 reference; allele frequency; variant:total alleles** | **EVS alleles; frequency** | **ExAc v.0.3.1 alleles; allele frequency** |
| --- | --- | --- | --- | --- | --- |
| *KLK4* | c.632delT | chr19:51410323 | rs556734208; 7.99 x10^-4^;  4/5008; all heterozygous | 1/6259*; 1.60 x10^-4^; all heterozygous | 2/120304; 1.66 x10^-5^; all heterozygous |

**Supplementary Table 8.** *KLK4* c.632delT allele frequency in databases of variation. Databases accessed 7^th^ September 2016. * variant is listed in EVS at position chr19:51410322 due to the EVS nomenclature system. Note that the variant is also reported in ClinVar as pathogenic: SCV000342116.2.

|  | **Threshold of calibrated enamel mineral density / g/cm^3^** | | |
| --- | --- | --- | --- |
|  | **≥2.13** | **2.13-2.75** | **≥2.75** |
| **Tooth** | **Mean enamel mineral density measurement for region selected by threshold (Supplementary Figure 3)** | | |
| control | 2.93 | 2.47 | 3.04 |
| KLK4 | 2.64 | 2.58 | 2.92 |

**Supplementary Table 9.** Mean enamel mineral density measurements for regions of enamel selected by thresholding.

| **Aspect** | Buccal | Buccal | Buccal | Buccal | Lingual | Lingual | Lingual | Lingual |
| --- | --- | --- | --- | --- | --- | --- | --- | --- |
| **Tooth** | Control | Control | KLK4 | KLK4 | Control | Control | KLK4 | KLK4 |
| **Layer** | Outer | Inner | Outer | Inner | Outer | Inner | Outer | Inner |
| **Readings** | 10 | 10 | 5 | 5 | 21 | 10 | 5 | 5 |
| **O** | 45.64 ± 0.86 | 45.70 ± 0.62 | 41.41 ± 2.19 | 46.85 ± 0.95 | 41.47 ± 1.40 | 46.64 ± 0.37 | 45.85 ± 2.94 | 39.21 ± 1.09 |
| **Ca** | 16.78 ± 0.39 | 18.05 ± 0.31 | 16.23 ± 0.64 | 15.12 ± 0.11 | 15.04 ± 0.91 | 17.91 ± 0.34 | 14.62 ± 1.14 | 13.16 ± 0.31 |
| **P** | 12.10 ± 0.14 | 12.48 ± 0.14 | 10.47 ± 0.50 | 9.95 ± 0.05 | 10.01 ± 0.60 | 12.13 ± 0.11 | 9.80 ± 0.73 | 8.38 ± 0.24 |
| **C** | 14.99 ± 0.58 | 13.54 ± 0.49 | 22.89 ± 3.27 | 17.52 ± 0.76 | 24.09 ± 3.10 | 13.58 ± 0.47 | 20.70 ± 5.07 | 26.10 ± 1.17 |
| **N** | 7.46 ± 0.43 | 7.17 ± 0.24 | 6.78 ± 0.13 | 8.20 ± 0.68 | 6.71 ± 0.27 | 7.02 ± 0.22 | 6.62 ± 0.35 | 10.99 ± 0.64 |
| **F** | 1.97 ± 0.11 | 1.90 ± 0.11 | 1.58 ± 0.09 | 1.45 ± 0.07 | 1.64 ± 0.11 | 1.78 ± 0.07 | 1.63 ± 0.15 | 1.36 ± 0.06 |
| **Na** | 0.90 ± 0.03 | 0.97 ± 0.04 | 0.50 ± 0.04 | 0.70 ± 0.08 | 0.70 ± 0.04 | 0.94 ± 0.04 | 0.62 ± 0.07 | 0.62 ± 0.05 |
| **Mg** | 0.17 ± 0.02 | 0.20 ± 0.02 | 0.17 ± 0.01 | 0.21 ± 0.03 | 0.11 ± 0.02 | 0.18 ± 0.03 | 0.16 ± 0.03 | 0.18 ± 0.02 |

**Supplementary Table 10.** EDX analysis of control and KLK4 enamel of incisor teeth. All readings are atomic mass percentage. The error is calculated as 2x standard error of the mean.

**Supplementary Videos.** Grey scale images of both canine and molar teeth from control individuals and family 1 V:1 KLK4 teeth. Video 1: control canine. Video 2: KLK4 canine. Video 3: control molar. Video 4: KLK4 molar.
